# COMPARATIVE ASSESSMENT OF TOPICAL STEROIDS, TOPICAL TRETENOIN (0.05%) AND DITHRANOL PASTE IN ALOPECIA AREATA

**DOI:** 10.4103/0019-5154.62747

**Published:** 2010

**Authors:** Sudip Das, R C Ghorami, T Chatterjee, Gautam Banerjee

**Affiliations:** *Bankura Sammilani Medical College, Bankura, West Bengal, India*; 1 *IPGMER, Kolkata, India.*

**Keywords:** *Alopecia areata*, *anthralin*, *topical steroids*, *tretenoin*

## Abstract

**Background:**

There have been various controversial reports regarding the efficacy of topical agents in topical therapy of alopecia areata.

**Aim:**

The study aims to find out the effective ones among the readily available ones for a dermatologist.

**Materials and Methods:**

Eighty patients were chosen from the skin OPD of Bankura Sammilani Medical College, Bankura, West Bengal, after evaluating the exclusion criterions. Treatments were continued for 3 month period and a follow up after further 3 months. After dividing them into four groups–group-I (topical steroids), group-II (topical tretinoin 0.05%) group-III (dithranol paste 0.25%), and group-IV (white soft petrolatum jelly)–patients were evaluated.

**Results:**

Seventy percent of group-I, 55% of group-II, 35% of group-III, and 20% of the control group (white soft petrolatum jelly) responded favorably. Side effects in the form of dermatitis and hyperpigmentation were seen in group-III. However, no patient discontinued from the study.

**Conclusion:**

We conclude that both topical steroids and tretinoin were fairly effective in limited variant of alopecia areata.

## Introduction

Alopecia areata is a non- scarring, recurrent, sometimes treatment-refractory hair disease, which can potentially cause hair loss in any hair bearing area. Alopecia areata commonly presents as patches of hair loss, of all scalp hair, (alopecia totalis), loss of body hair (alopecia universalis), or an ophiasis (band like) pattern. Common diseases associated with alopecia areata include allergic rhinitis, asthma, atopic dermatitis, and thyroid disorders.[1] It has also been associated with other diseases, most of which are autoimmune in nature e.g. vitiligo, lupus erythematosus, rheumatoid arthritis, pernicious anemia, scleroderma, ulcerative colitis, and diabetes mellitus.

In early active alopecia areata, the hair cycle is disrupted and expected ratio of anagen, catagen, and telogen for the site is abnormal, with most hair follicles entering the telogen phase or late categen phase.[2] There is no ‘best’ treatment for alopecia areata and no strong evidence to suggest that any drug induce remissions or therapies alter the course of alopecia areata and is believed that available modes of treatment alter or suppress the underlying process. Many treatments, both topical and parentral, are available, however efficacy, risk, and benefits of each treatment have to be considered before choosing a treatment.

Topical steroids are frequently used to treat limited alopecia areata and in some cases extensive alopecia areata. Anthralin and tretinoin are irritants and induce hair growth by unknown mechanisms, we therefore tried to evaluate the three easily available topical agents-topical steroids (betamethasone dipropinate), tretinoin (0.05%) cream, and dithranol paste to find out the best and most effective topical therapy for limited variant of alopecia areata.[3]

## Materials and Methods

A total number of 80 consecutive patients reporting to the skin OPD of Bankura Sammilani Medical College, Bankura, were included in the study. They were divided into four groups (each of 20 patients)–group-I was treated with topical steroids. (betamethasone dipropionate lotion), group-II with tretinoin 0.05% cream, group-III with dithranol paste 0.25%, and group-IV with white soft petrolatum jelly. The medicaments were applied twice daily for 3 months and results were noted thereafter further 3 months. Exclusion criteria of patients include (a) trichotillomania; (b) hypogonadism; (c) hair shaft disorders; (d) patches >5 cm diameter; (e) more than five patches; (f) alopecia totalis, subtotalis, and universalis; (g) triangular alopecia and ophiasic pattern of alopecia. Trichogram was done in each patient after clinical assessment.

About 30-50 hairs were clipped and seen under a 10× microscope. 10% KOH examination was done to look for dermatophytes in all patients to rule out any possibility of fungal infection. Clinical history was taken to rule out the presence of atopy or patchy loss of hair, thyroid disorders, vitiligo, diabetes in oneself or other members of family. Changes (if any) were noted and clinical assessment of other hair bearing parts of the body, such as beard, moustache, were noted.

Patients were *followed up* every 4 weeks. Hair count and clinical assessment were done at baseline (0 weeks), 4, 8, and 12 weeks. If by 12th week, 60% of hair density is seen compared to normal on similar areas, we labeled them as good growth provided hair count show significant growth. We observed the patients for further 3 months at 1 month interval to see any loss of hair.

## Results

The youngest patient reporting to us was of 6 year and oldest was of 44 years. The mean age of the patient was 28 years. Atopy was present in 17 of the 80 patients (21.2%). Family history of patchy loss of hair was seen in 6 out of 80 patients (7.5%). History of diabetes was seen in 7 out of 80 patients (8.75%), vitiligo in 6 out of 80 patients (7.5%), thyroid disorder in 3 out of 80 patients (3.75%) and nail changes were noted in one-third of the patients studied.

Regrowth of hair was seen in 3 month time in 70% of patients of group-I. In group-II (Tretinoin 0.05%) 55% of patients showed good regrowth (11 out of 20). In group-III (Dithranol paste 0.25%) 35% of the patient showed good regrowth (7 out of 20) and in group-IV 20% showed good regrowth within (4 out of 20) 3 month time. Trichogram was done in all patients and 70 out of 80 [Table 1] patients did show abundance of telogen hairs with few catagen hairs 10% KOH examination was negative for dermatophytes in all patients. No side effects except for mild irritation and hyperpigmentation were noted in 100% of patients of group-III.

**Table 1:** Regrowth of hairs with various topical therapies

Groups	No. of patients	Good regrowth	Percentage	Adverse effects
GRI (Steroids)	20	14	70	NIL
GRII (Tretenoin 0.05%)	20	11	55	NIL
GRIII (Dithranol 0.25%)	20	07	35	Hyperpigmentation and dermatitis
GRIV (Petrolatum jelly)	20	04	20	NIL

## Discussion

Our results showed a significant hair growth in the topical steroid group (70%) and tretinoin group (35%). Dithranol paste applied topically showed reasonably good response in more than one-third of patients. But side effects, such as irritation, hyperpigmentation, were present only in group–III. In group–IV, 20% patients showed good regrowth in 3 month time justifying that spontaneous regrowth occurs in alopecia areata. *Regrowth in our study was defined as more than 60% coverage of the bald patch in 3 months time.*

Montes *et al*.[4] using 0.1% halocinonide cream applied twice daily with or without occlusion reported 100% regrowth in all patients in 6 to 18 months of treatment. Pascher *et al*.[5] showed excellent (total to near total) response with 0.2% flucinonide acetate in 17 of 28 patients. They also reported that this mode was more effective in children compared to adults. Topical tretinoin in a study by Baird *et al*.[6] showed insignificant response when applied for 3 month period. Very few studies are available for the efficacy of topical tretinoin in alopecia areata, but our study did show a reasonable good response in 55% of our patients.

Topical anthralin was used by Schmoeckel *et al*.[7] and 18 of 24 patients showed regrowth but incidents of side effects (dermatitis, hyperpigmentation, and lymphadenopathy) were noted. Another study by Fielder Weiss *et al*.[8] showed 50% regrowth, but side effects were high. As already reported, one-fourth of limited variant of alopecia areata showed good regrowth in our study that is inconformity with most other studies available.

Our study therefore proves that topical steroid is still the most effective therapy for limited variant of alopecia areata but topical tretinoin does showed a good response. Topical anthralin shows fair response but side effects are more. We are not sure, whether combinations of topical tretinoin and steroids are more superior than agents used alone, and a further study is being planned to check if they are more effective.
